# Supplementary material for: 2-Alkyl-Substituted-4-Amino-Thieno[2,3-d]Pyrimidines: Anti-Proliferative Properties to In Vitro Breast Cancer Models
Source: Molecules. 2023 Aug 30;28(17):6347. doi: 10.3390/molecules28176347 (PMC10489817; doi:10.3390/molecules28176347)
Supplement: Supplementary file 1 [file molecules-28-06347-s001.zip › molecules-2565955-supplementary.pdf]

Supplementary Materials

2-Alkyl-Substituted-4-Amino-thieno[2,3-*d*]pyrimidines: Anti-proliferative Properties to *in vitro* Breast Cancer Models

Ivan Iliev[1#], Anelia Mavrova[2#], Denitsa Yancheva[3], Stefan Dimov[2], Galya Staneva[4], Alexandrina Nesheva[4], Iana Tsoneva[4*], Biliana Nikolova[4*]

1 Institute of Experimental Morphology, Pathology and Anthropology with Museum, Bulgarian Academy of Sciences, Acad. G. Bonchev Str., bl.25, 1113 Sofia, Bulgaria; taparsky@abv.bg

2. University of Chemical Technology and Metallurgy, 8 Kliment Ohridski Blvd., 1756 Sofia, Bulgaria; anmav@abv.bg (A.M.); s_t_e_v_e@abv.bg (S.D.)

3 Institute of Organic Chemistry with Centre of Phytochemistry, Bulgarian Academy of Sciences, Acad. G. Bonchev Str., bl. 9, 1113 Sofia, Bulgaria; denitsa.pantaleeva@orgchm.bas.bg

4 Institute of Biophysics and Biomedical Engineering, Bulgarian Academy of Sciences, Acad. G. Bonchev Str., bl.21, 1113 Sofia, Bulgaria g_staneva@yahoo.com (G.S.), nescheva@gmail.com (A.N.)

Contents:

1. Scheme S1. Molecular geometry of compound 4, optimized at B3LYP/6-311++G level of theory in DMSO solvent, and atom numbering in the thienopyrimidine core**

……………………………………………………………………………………. p.2

2. Table S1. Chemical shifts predicted by GIAO method at B3LYP/6-311++G level of theory in DMSO solvent and experimental data for compound 4………. p. 3**

3. IR spectra (Figures S1-a,b – S4-a,b)……………………………………… p.4-p.7

4. NMR spectra (Figures S5-a,d-S8-a,d)……………………………...…… p.7-p.23

5.Cytotoxicity, Cell cycle arrest (Figures S9-S11)……………………………. p.24-p.25

6.Predicted protein targets (Figures S12-S14)……………..…………………. p.26-p.26

7. Calculated lipophilicity of compounds 2, 3, 4 and 5 (SwissADME)….. p.27

Scheme S1. Molecular geometry of compound **4**, optimized at B3LYP/6-311++G** level of theory in DMSO solvent, and atom numbering in the thienopyrimidine core

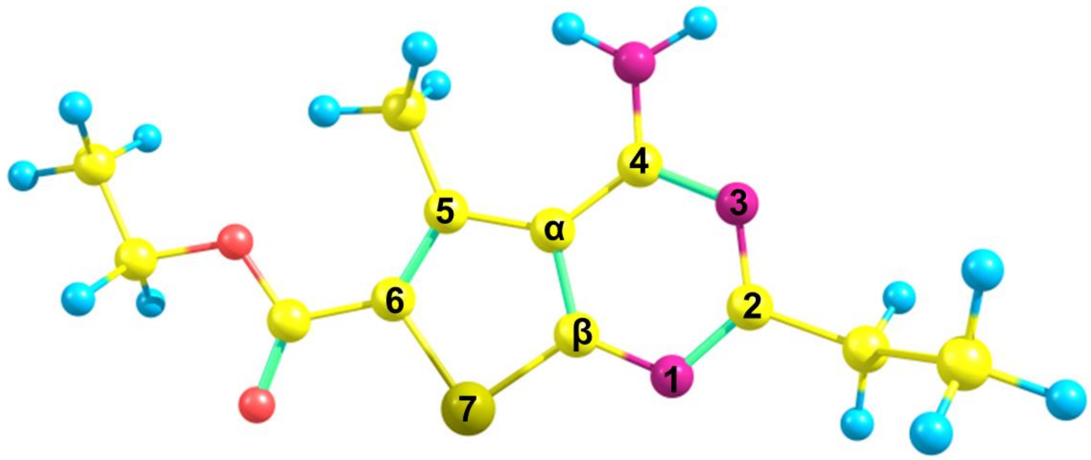

Table S1. Chemical shifts predicted by GIAO method at B3LYP/6-311++G** level of theory in DMSO solvent and experimental data for compound **4**

**Assignment to atoms[a]**	**GIAO/ DMSO**	**Calc. chem. shift/ DMSO**	***δ exp (DMSO)***	**Numbering in calc.**
		thienopyrimidine core:		
N1	-8.84	193.57	-	23
C2	4.47	180.26	168.36	22
N3	-0.69	185.42	-	21
C4	18.27	166.46	160.53	20
C5	35.19	149.54	140.82	1
C6	48.35	136.38	119.53	2
βC	64.50	120.22	114.65	3
S7	266.67	-81.95	-	5
αC	2.11	182.62	169.60	4
		2-ethyl group:		
$-\underline{C}H_2CH_3$	144.30	40.43	32.08	24
$-CH_2\underline{C}H_3$	166.83	17.90	14.62	30
$-C\underline{H}_2CH_3$	29.27	2.70	2.62-2.70 (q, J = 7.6 Hz. 2H)	25
$-C\underline{H}_2CH_3$	29.20	2.77	2.62-2.70 (q, J = 7.6 Hz. 2H)	26
$-CH_2C\underline{H}_3$	30.77	1.20	1.28-1.32 (t, J = 7.0 Hz. 3H)	31
$-C\underline{H}_2CH_3$	30.53	1.44	1.28-1.32 (t, J = 7.0 Hz. 3H)	32
$-C\underline{H}_2CH_3$	30.66	1.31	1.28-1.32 (t, J = 7.0 Hz. 3H)	33
		4-amino group:		
$-\underline{N}H_2$	160.13	24.60	-	27
$-N\underline{H}_2$	26.10	5.86	7.22-7.29 (bs, 2H)	28
$-N\underline{H}_2$	26.56	5.41	7.22-7.29 (bs, 2H)	29
		5-methyl group:		
$-\underline{C}H_3$	166.28	18.45	15.97	6
$-C\underline{H}_3$	27.74	4.23	2.84 (s. 3H)	7
$-C\underline{H}_3$	29.49	2.48	2.84 (s. 3H)	8
$-C\underline{H}_3$	29.47	2.49	2.84 (s. 3H)	9
		6-ethyl carboxylate group:		
$-\underline{C}=O$	1.11.2492	173.48	162.84	10
$-\underline{O}-C=O)$	-61.19	245.92	-	13
$-\underline{O}CH_2CH_3$	106.52	78.21	-	11
$-O\underline{C}H_2CH_3$	115.16	69.57	61.46	12
$-OCH_2\underline{C}H_3$	168.55	16.18	12.84	14
$-OC\underline{H}_2CH_3$	27.61	4.36	4.26-4.32 (q, J = 7.0 Hz. 2H)	15
$-OC\underline{H}_2CH_3$	27.58	4.39	4.26-4.32 (q, J = 7.0 Hz. 2H)	16
$-OCH_2C\underline{H}_3$	30.32	1.65	1.19-1.25 (t, J = 7.5 Hz. 3H)	17
$-OCH_2C\underline{H}_3$	30.58	1.39	1.19-1.25 (t, J = 7.5 Hz. 3H)	18
$-OCH_2C\underline{H}_3$	30.35	1.62	1.19-1.25 (t, J = 7.5 Hz. 3H)	19

[a] Numbering according to scheme 1

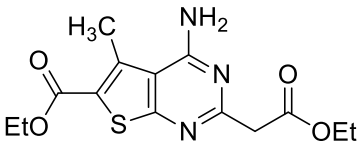

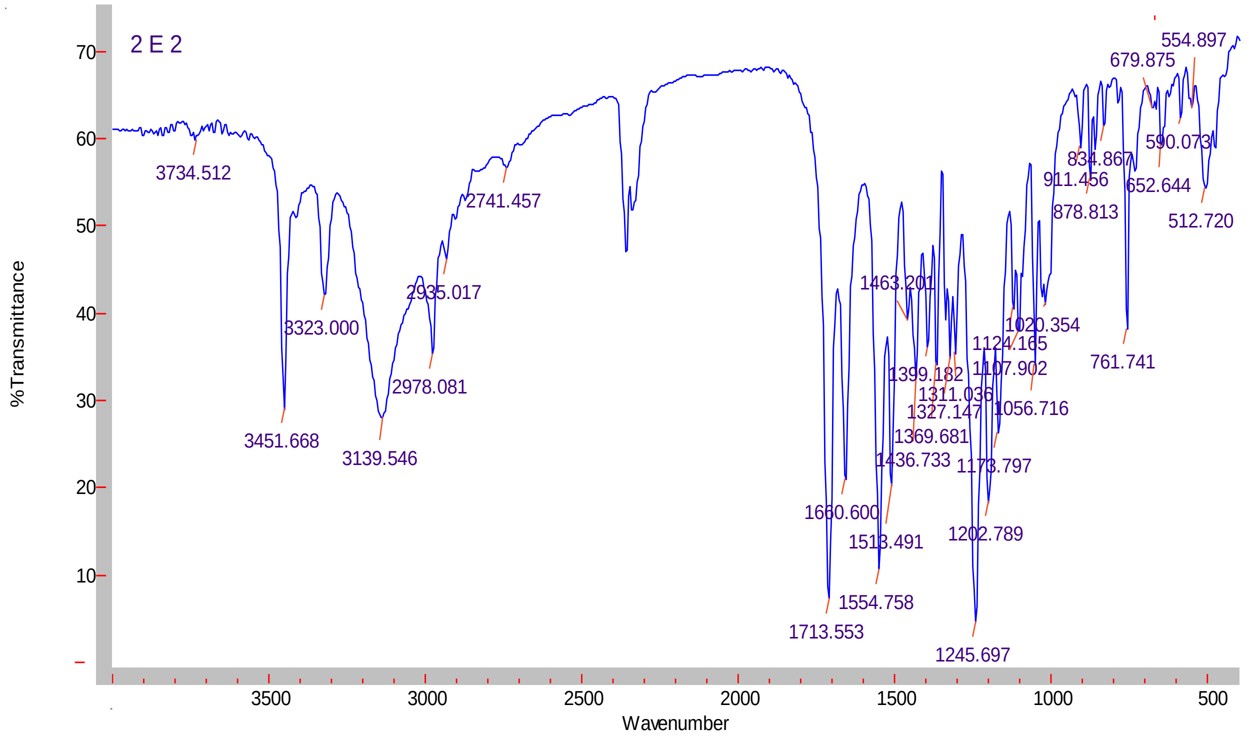

Figure S1-a IR spectrum of compound **2**

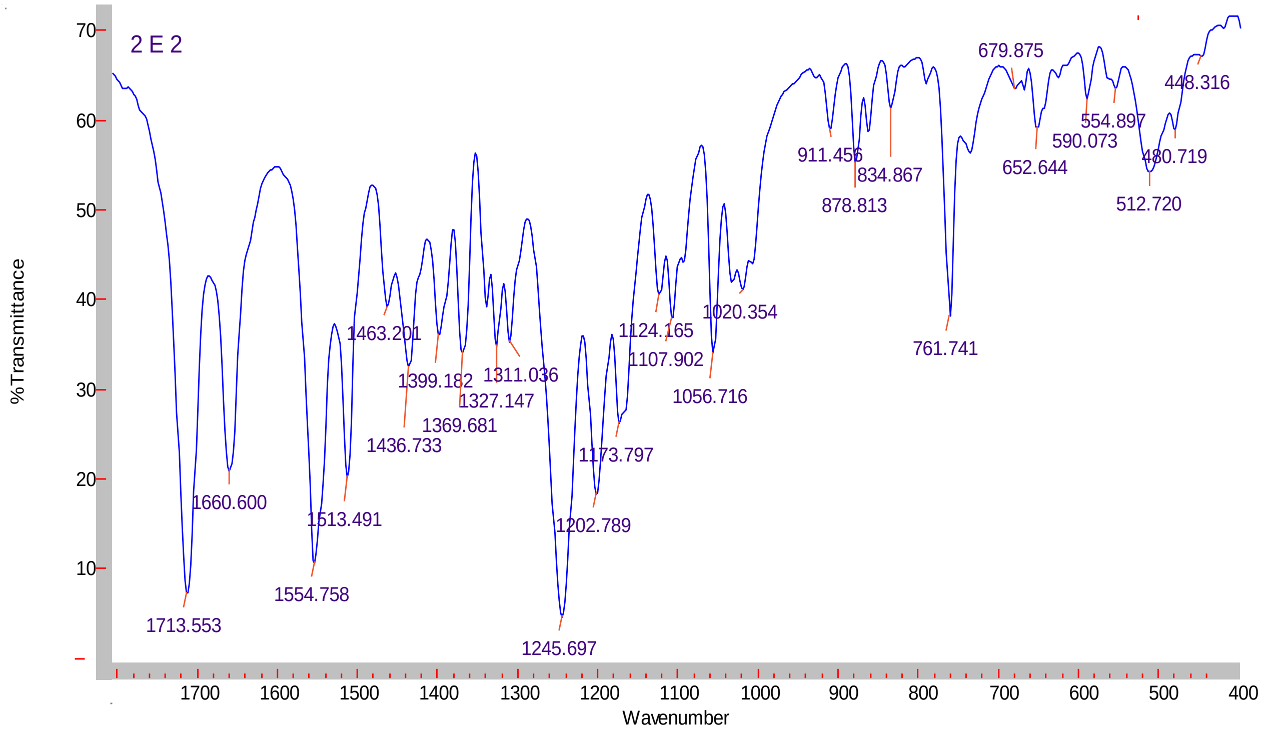

Figure S1-b IR spectrum of compound **2**

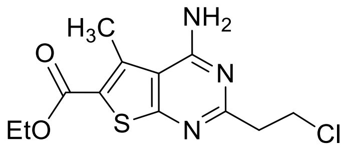

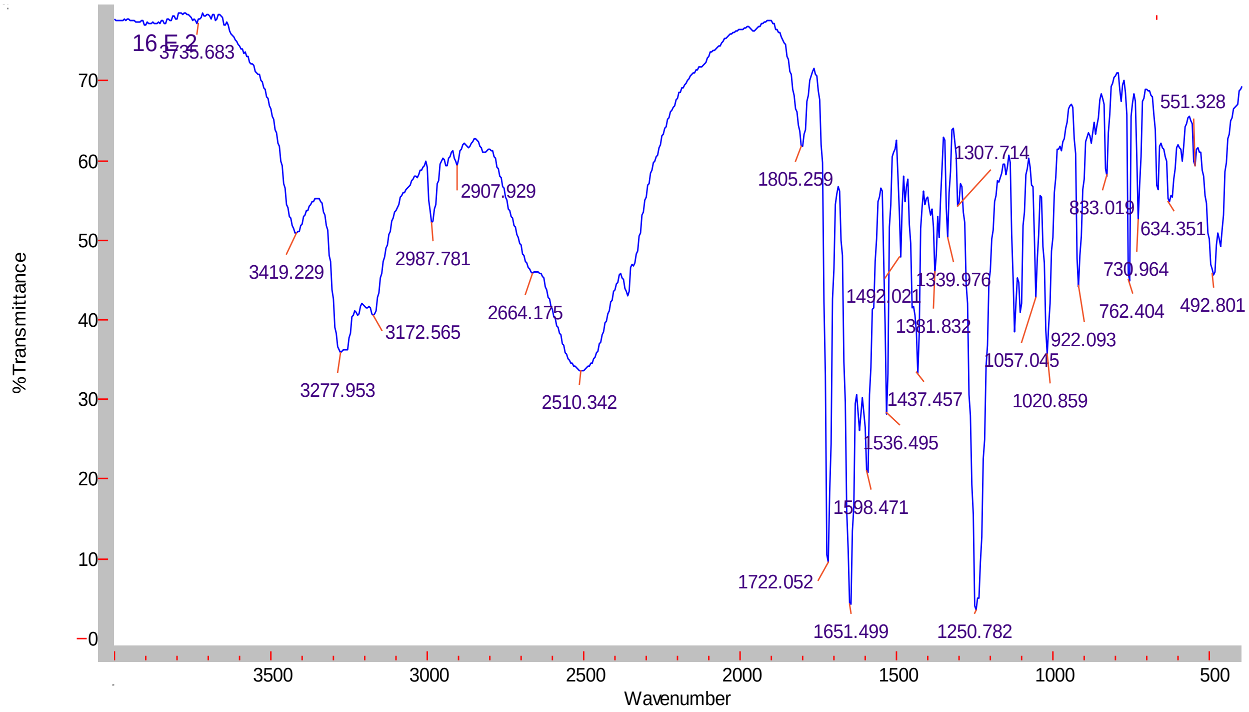

Figure S2-a IR spectrum of compound **3**

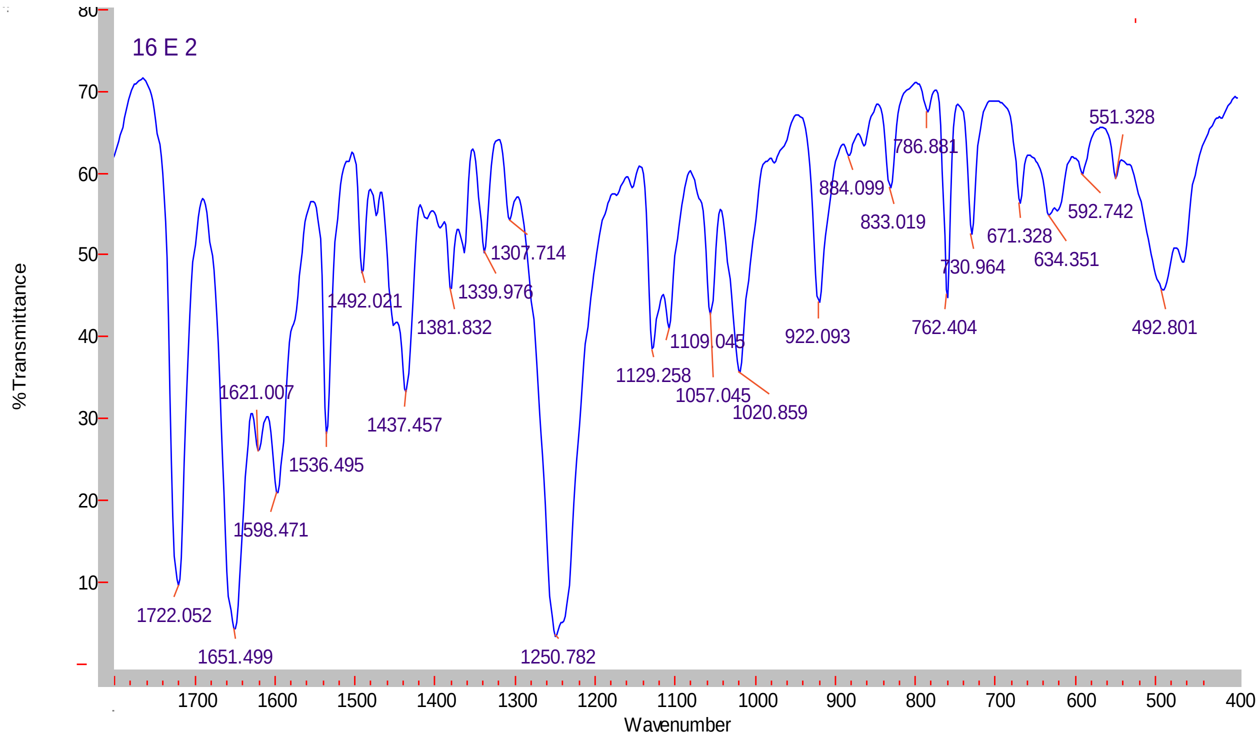

Figure S2-b IR spectrum of compound **3**

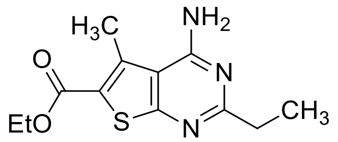

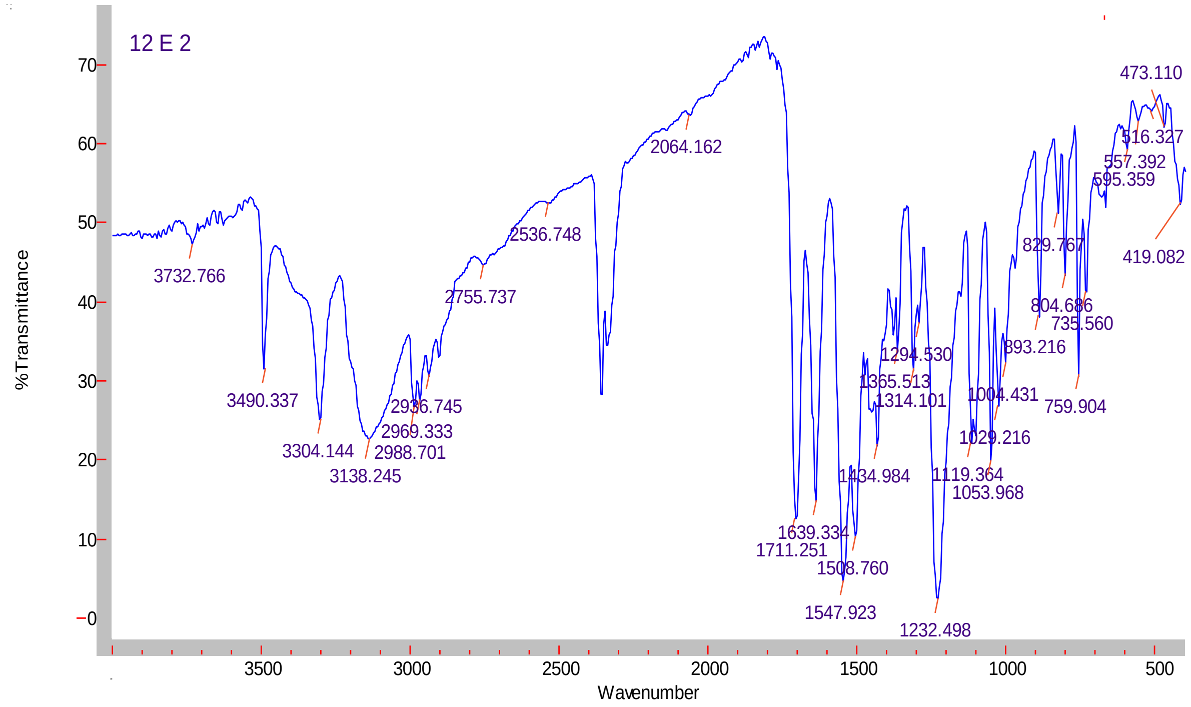

Figure S3-a IR spectrum of compound **4**

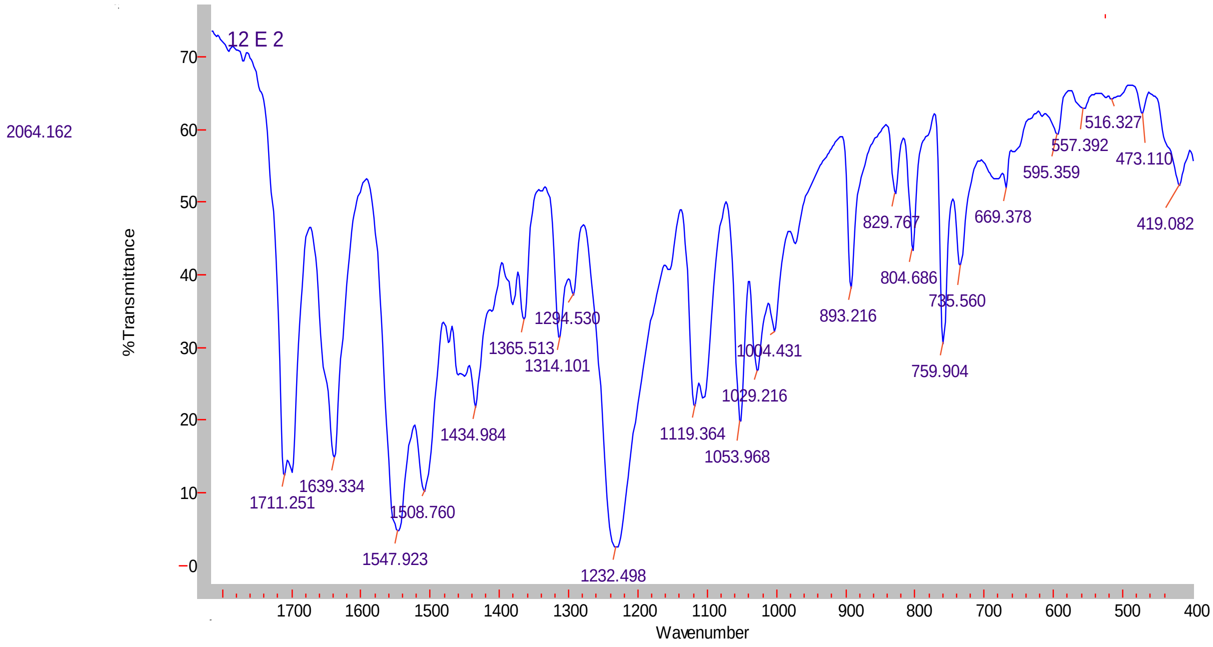

Figure S3-b IR spectrum of compound **4**

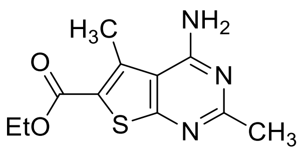

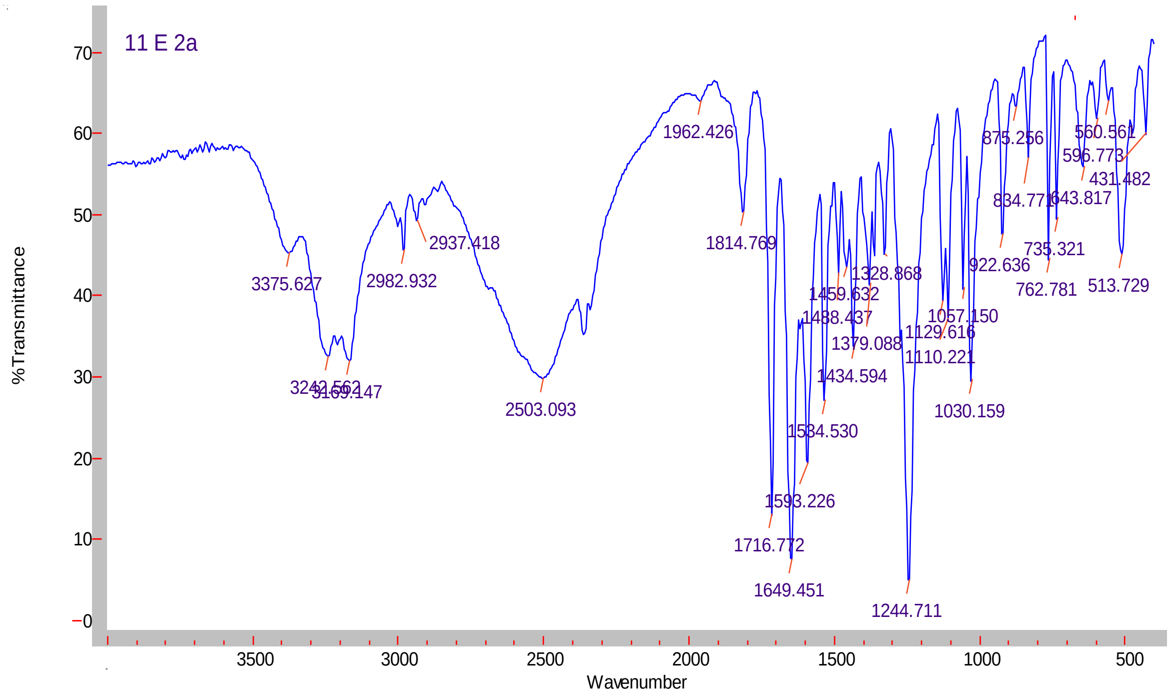

Figure S4-a IR spectrum of compound **5**

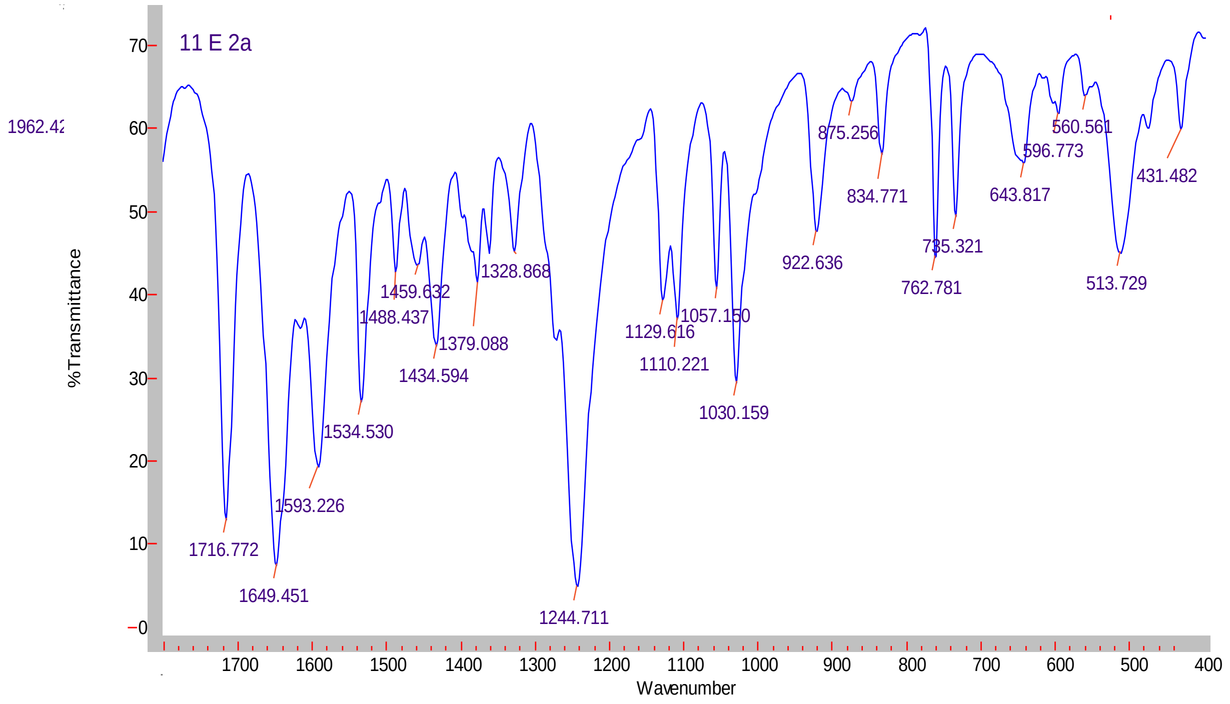

Figure S4-b IR spectrum of compound **5**

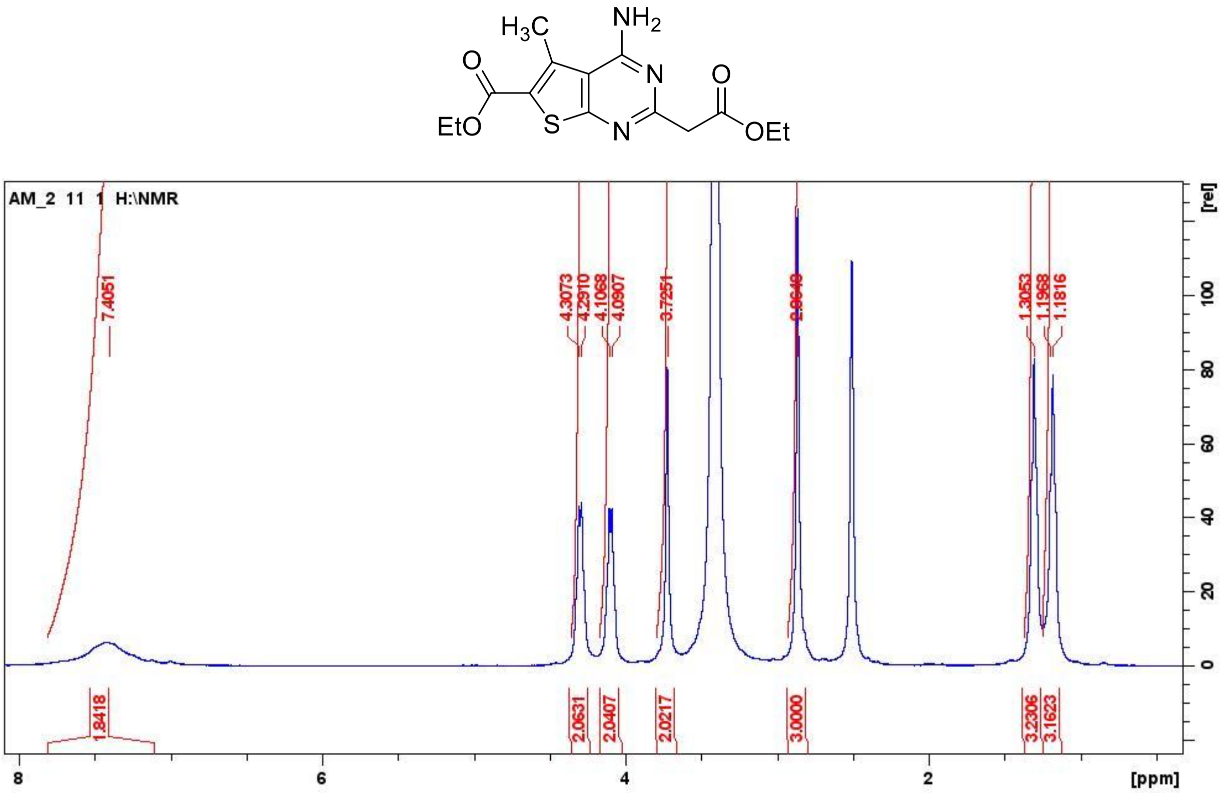

Figure S5-a ^{1}H NMR spectrum of compound **2** in DMSO-d_6

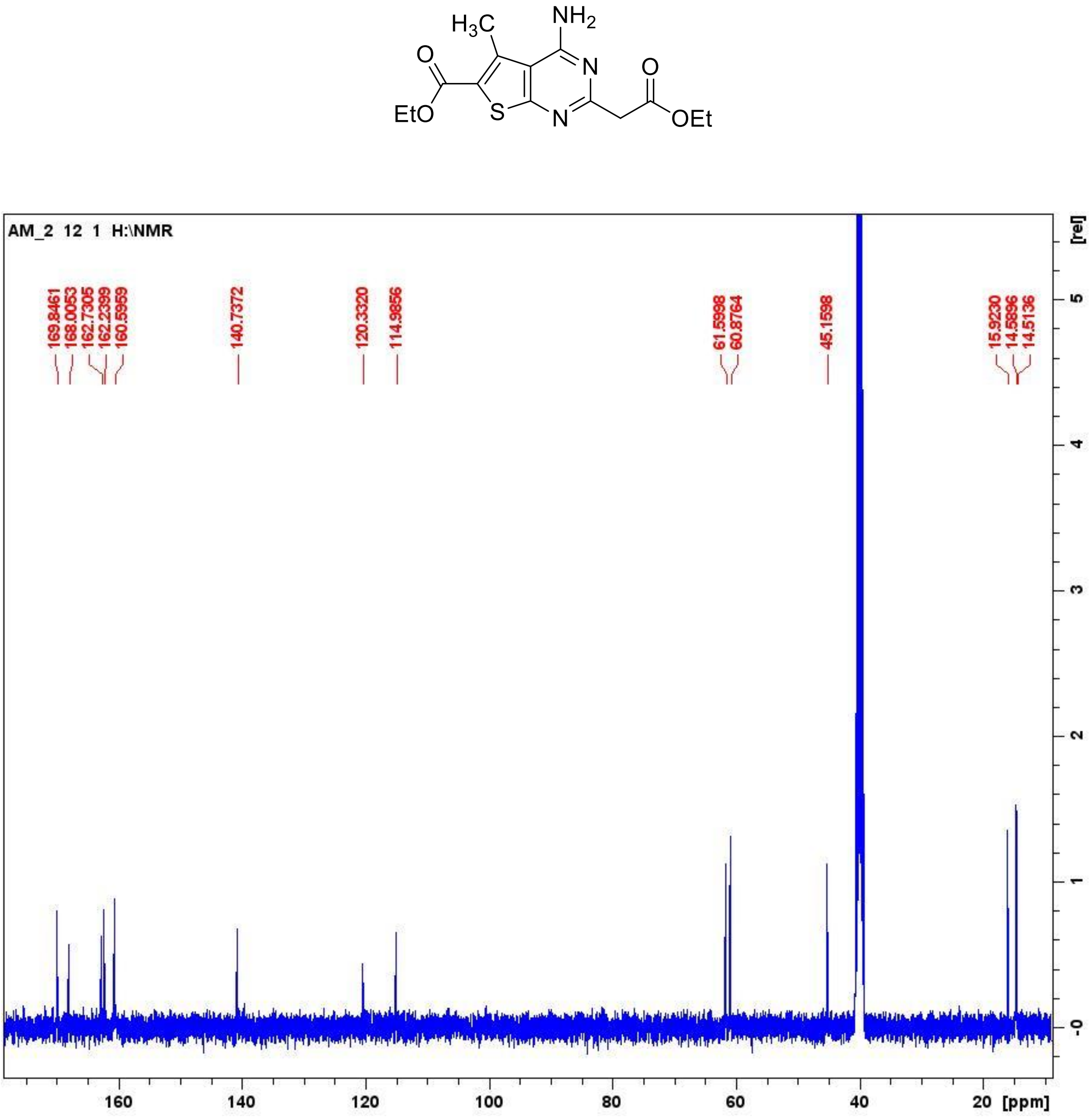

Figure S5-b ^{13}C NMR spectrum of compound **2** in DMSO-d_6

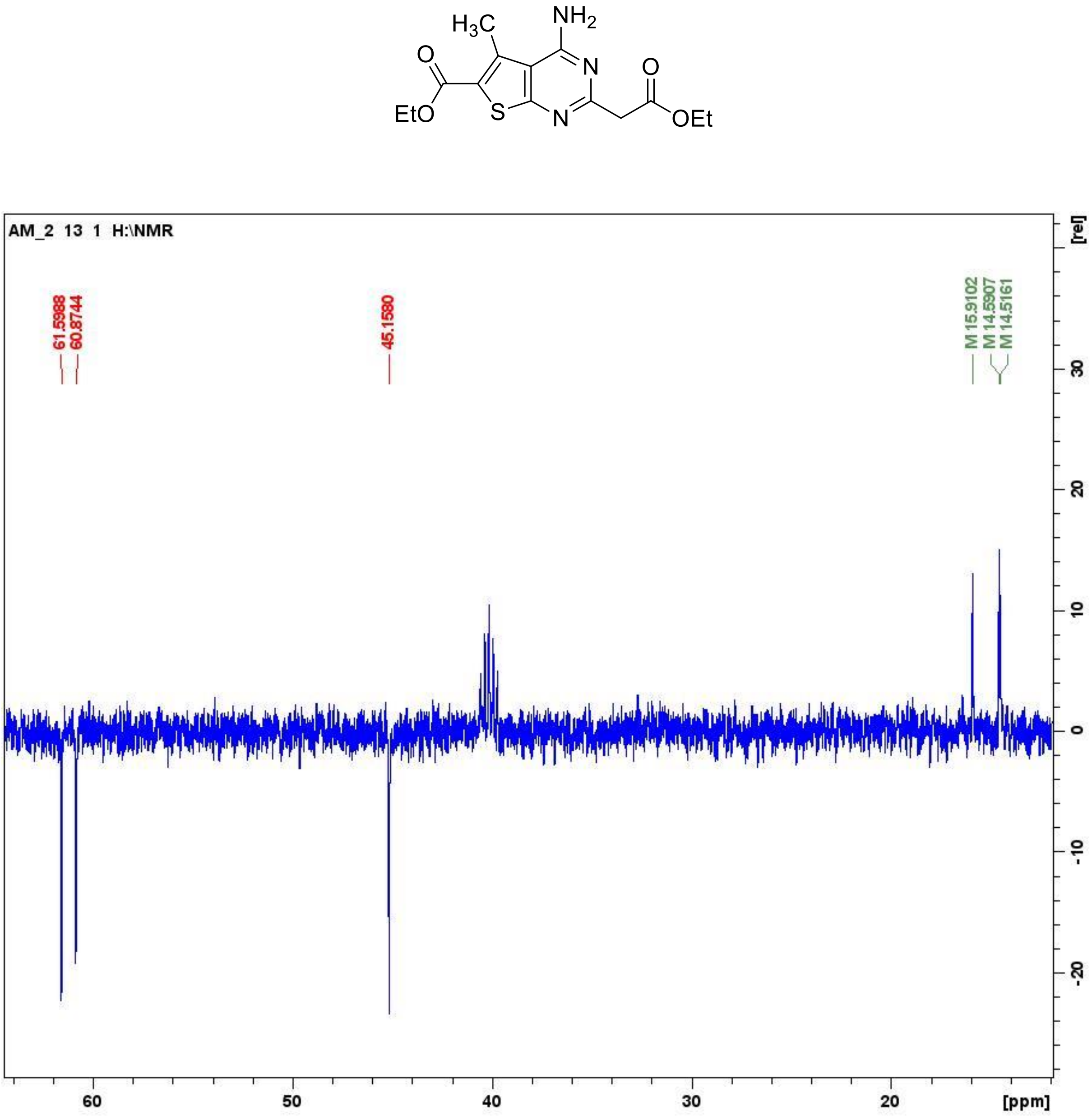

Figure S5-c DEPT-135 NMR spectrum of compound **2** in DMSO-d_6

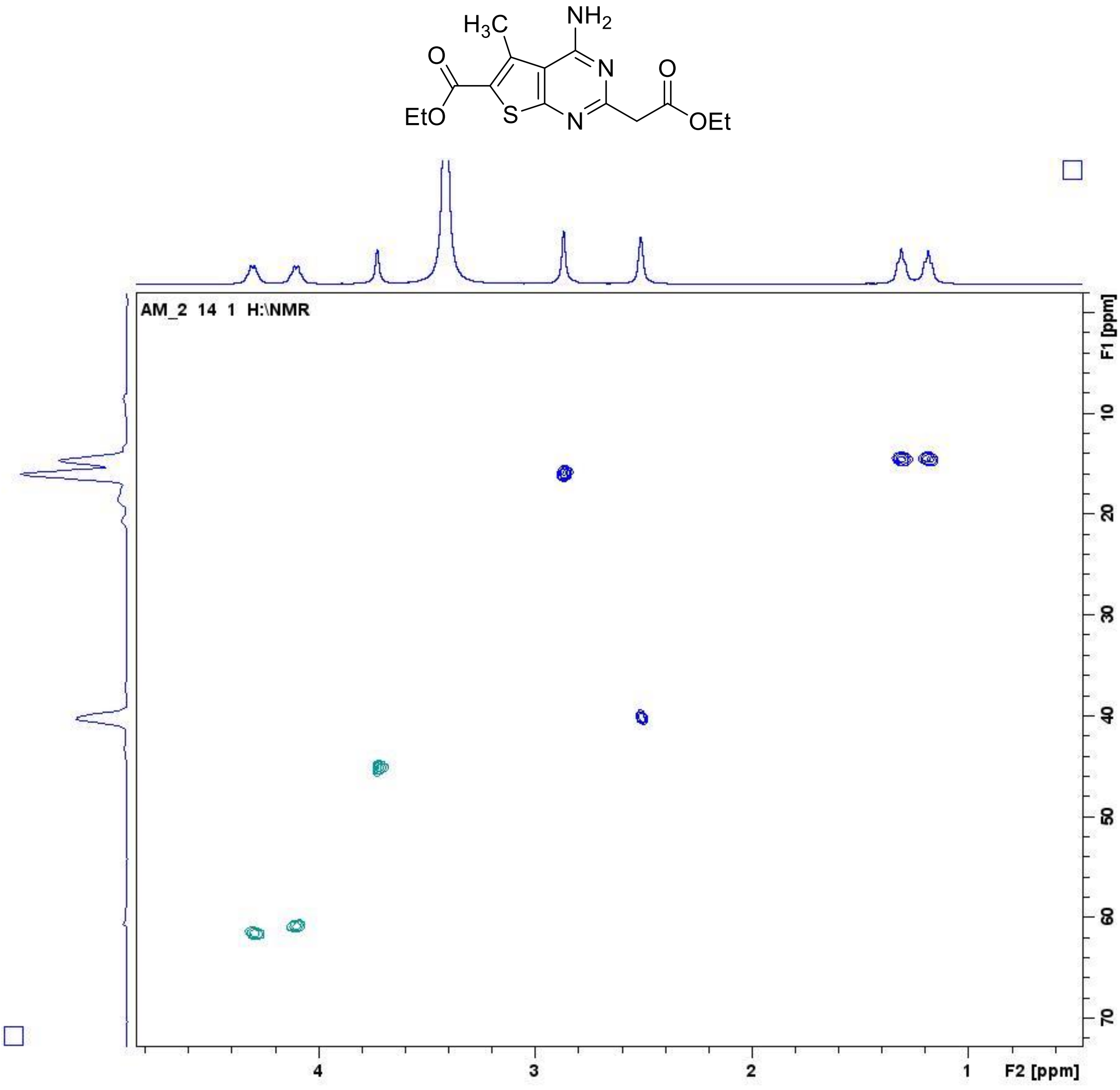

Figure S5-d HSQC NMR spectrum of compound **2** in DMSO-d_6

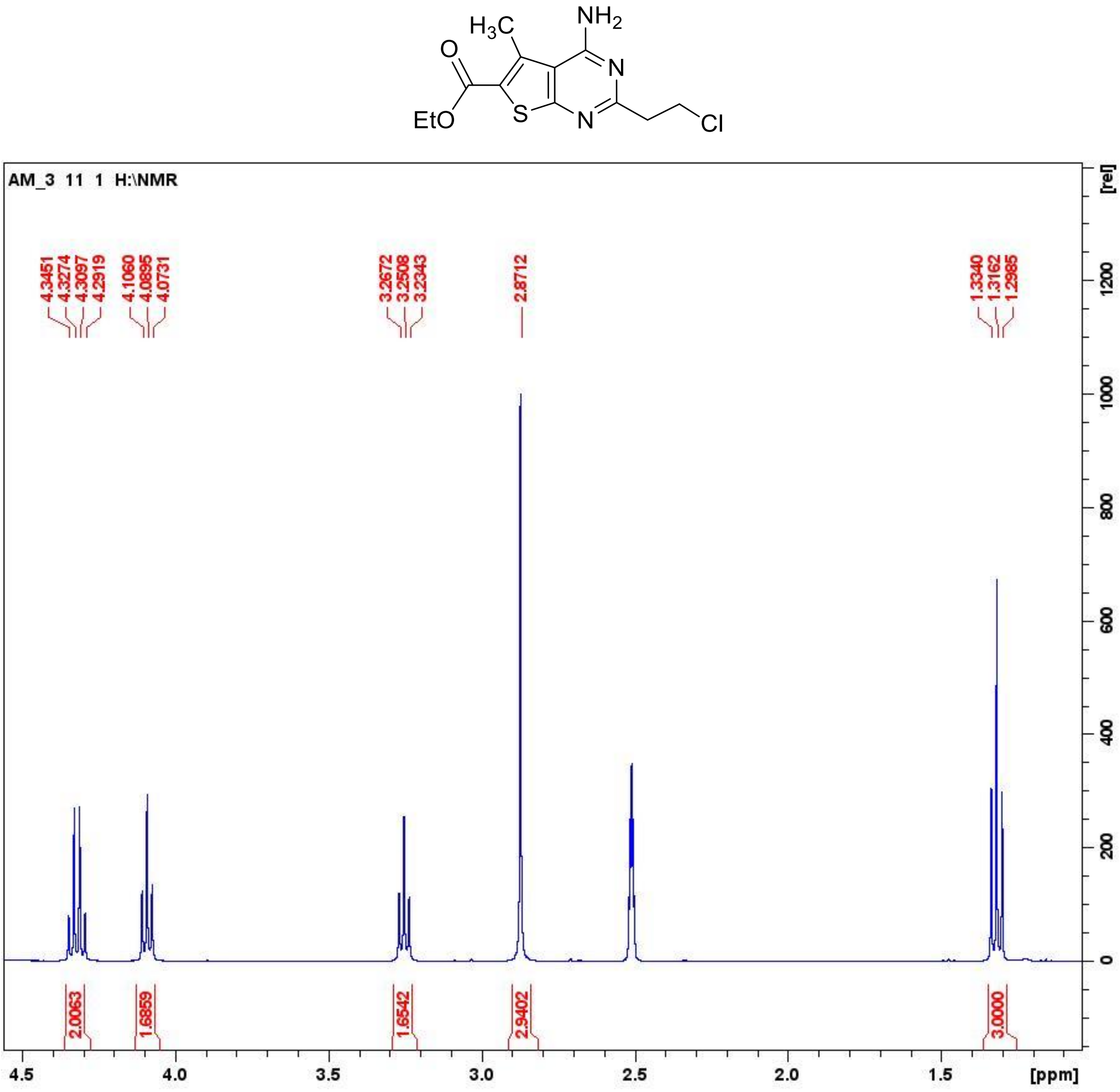

Figure S6-a ^{1}H NMR spectrum of compound **3** in DMSO-d_6

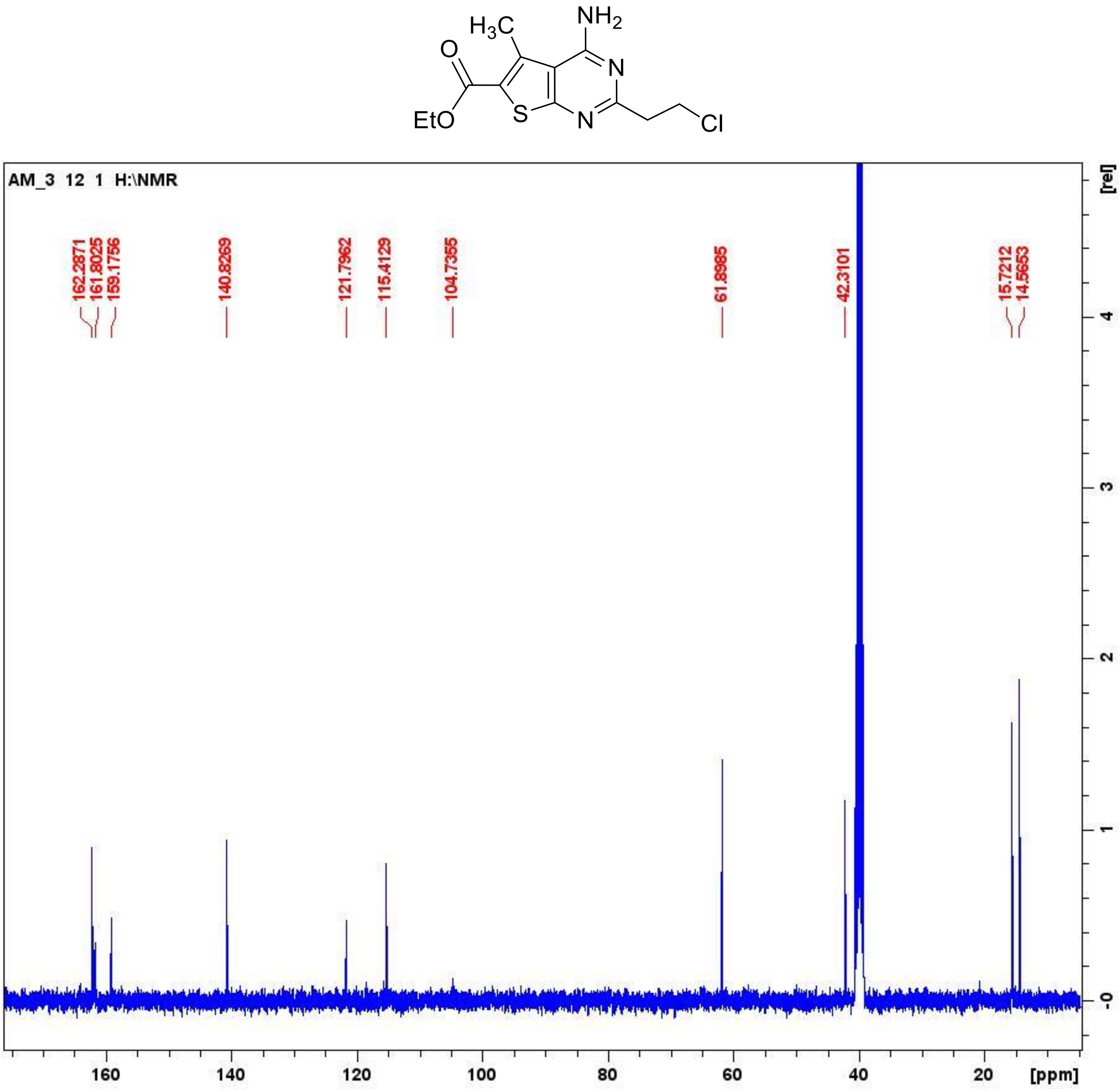

Figure S6-b ^{13}C NMR spectrum of compound **3** in DMSO-d_6

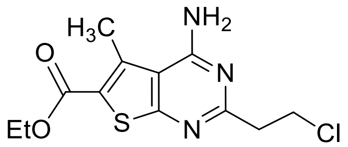

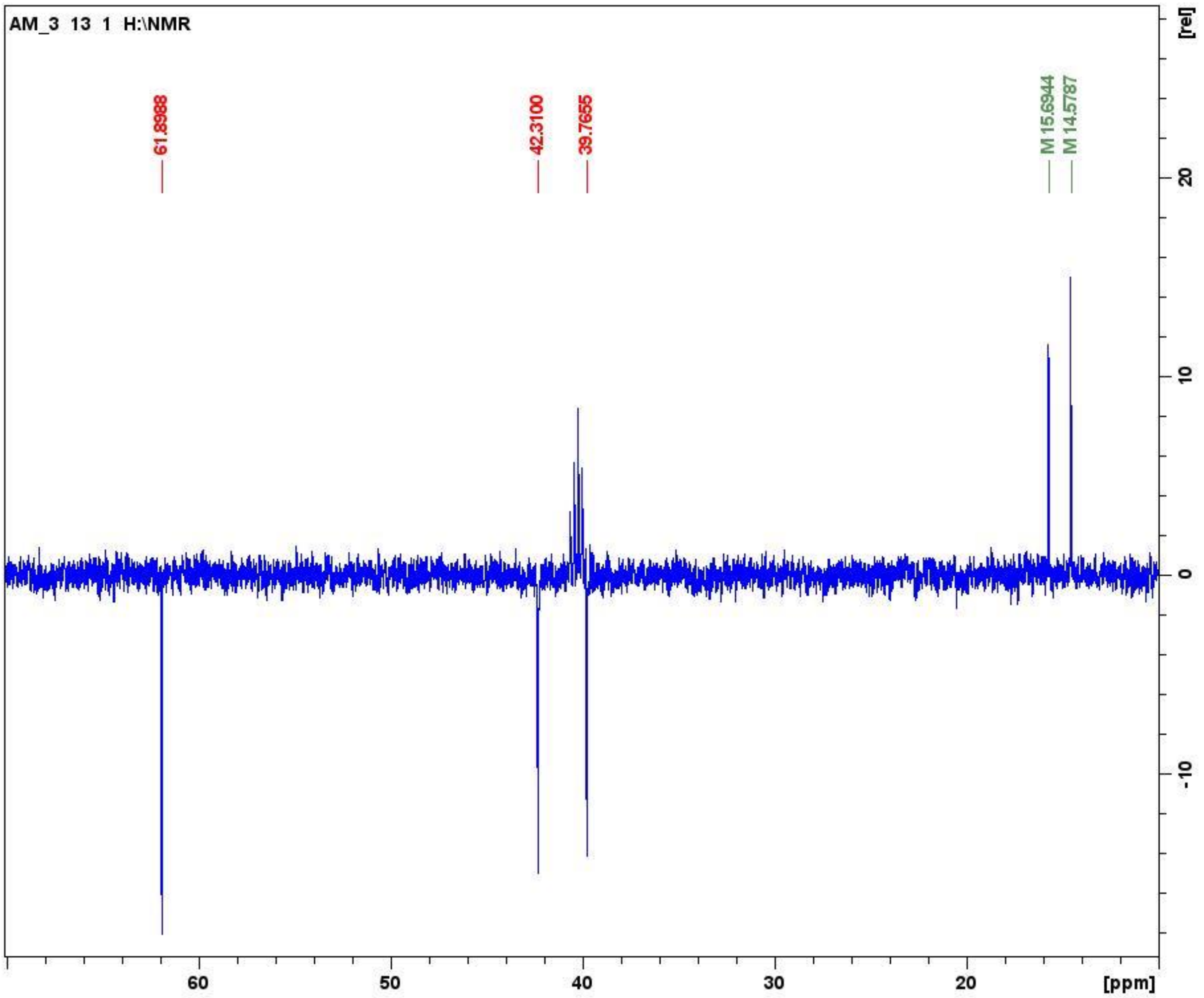

Figure S6-c DEPT-135 NMR spectrum of compound **3** in DMSO-d_6

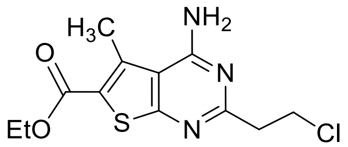

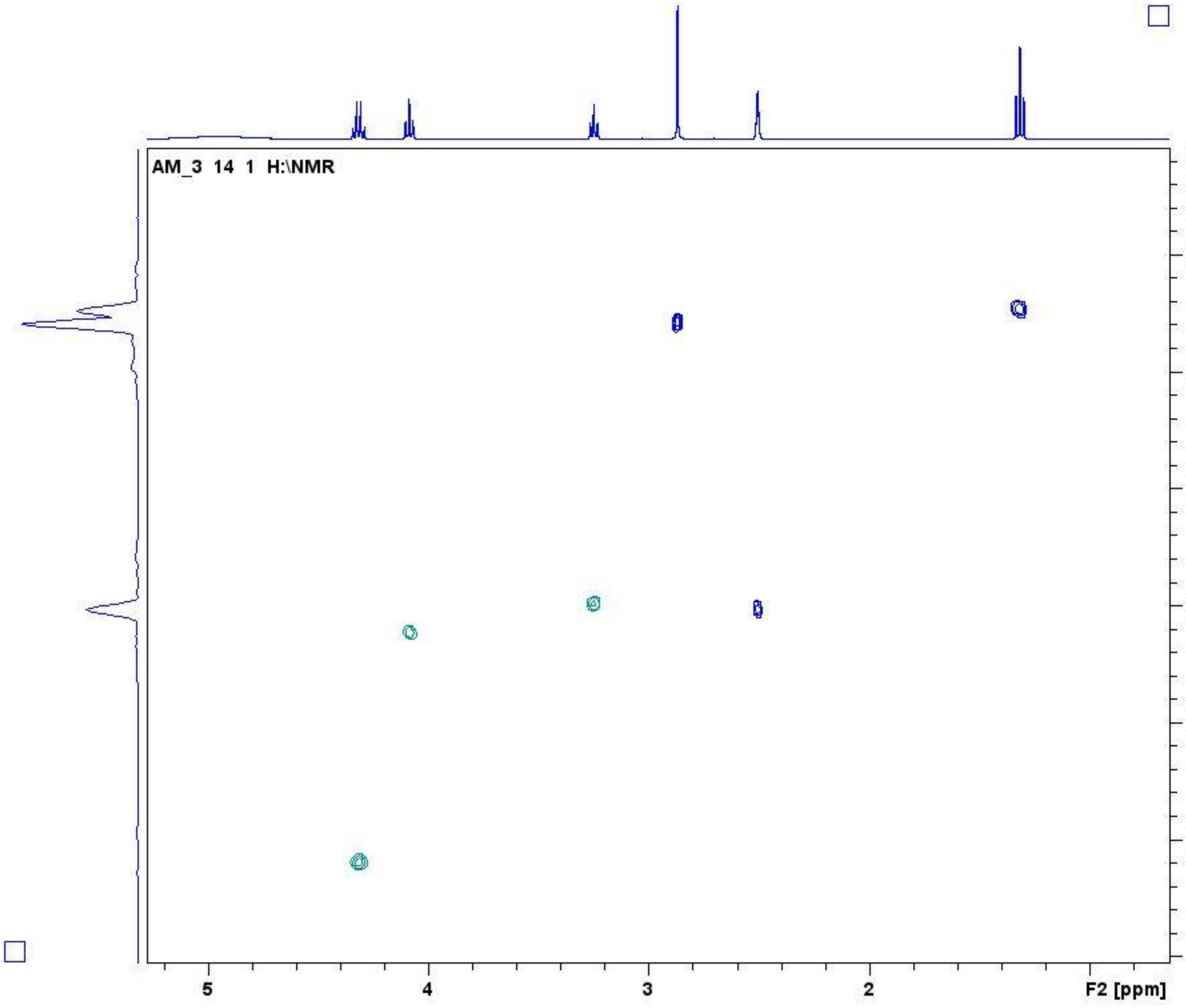

Figure S6-d HSQC NMR spectrum of compound **3** in DMSO-d_6

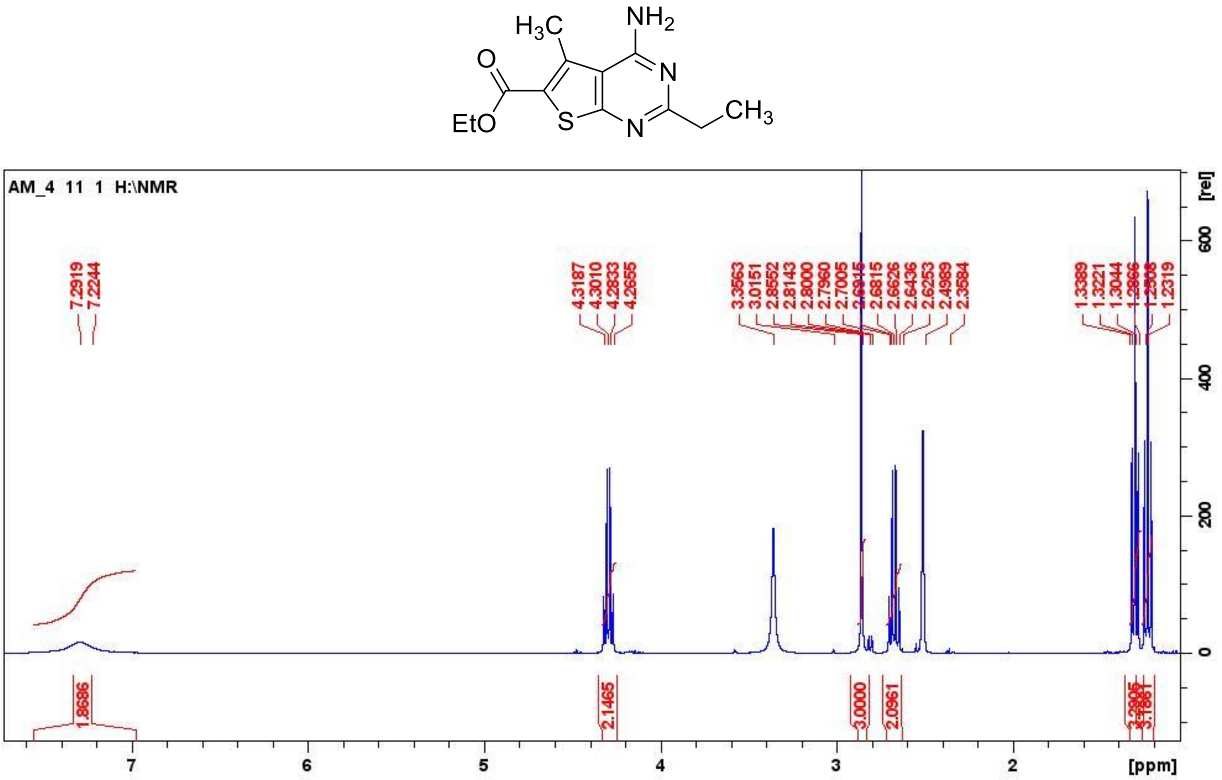

Figure S7-a ^{1}H NMR spectrum of compound **4** in DMSO-d_6

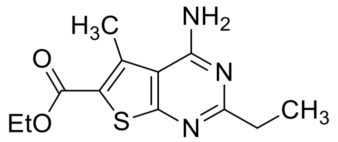

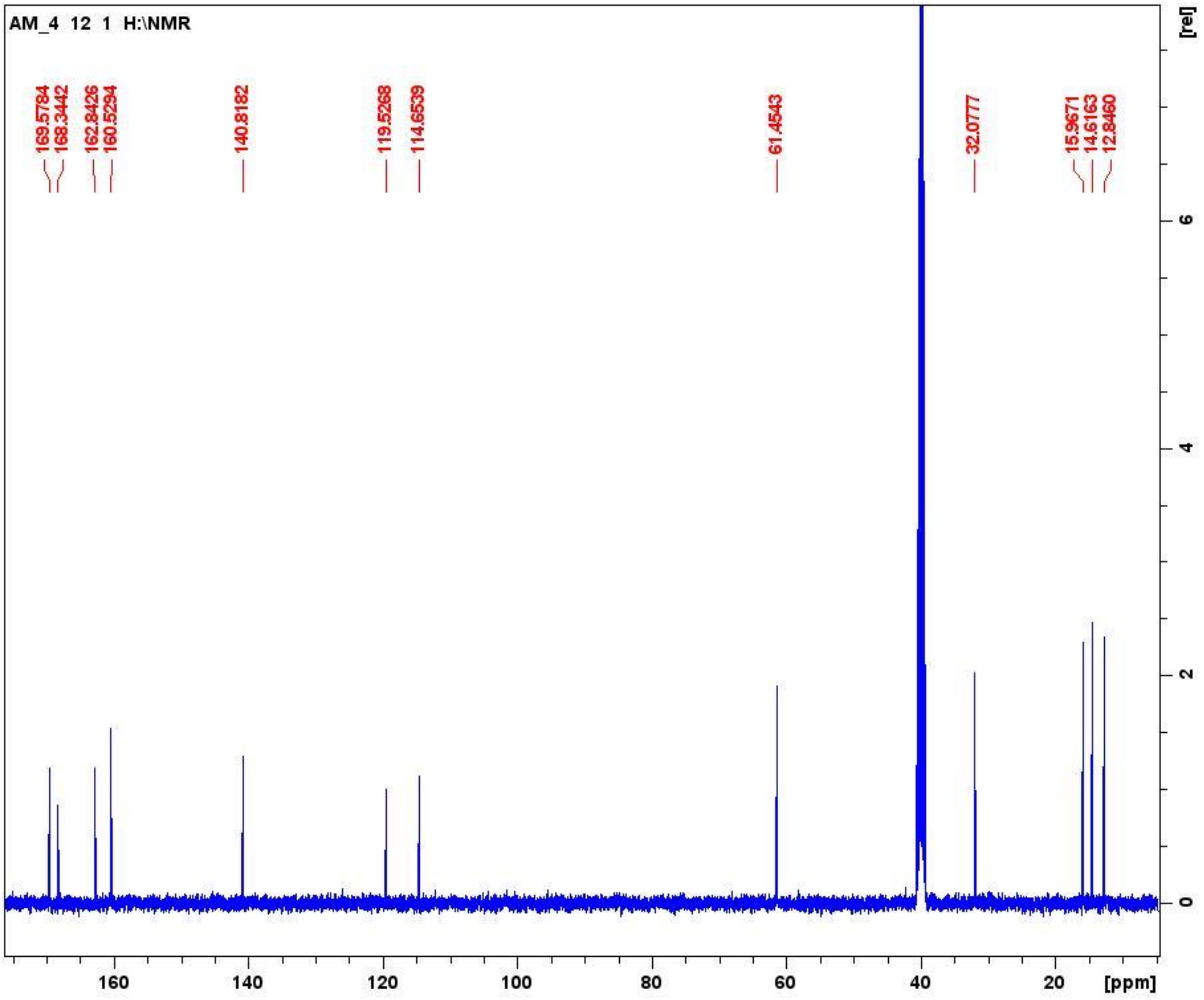

Figure S7-b ^{13}C NMR spectrum of compound **4** in DMSO-d_6

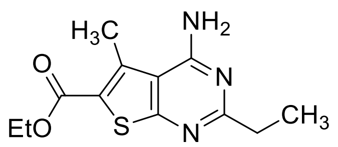

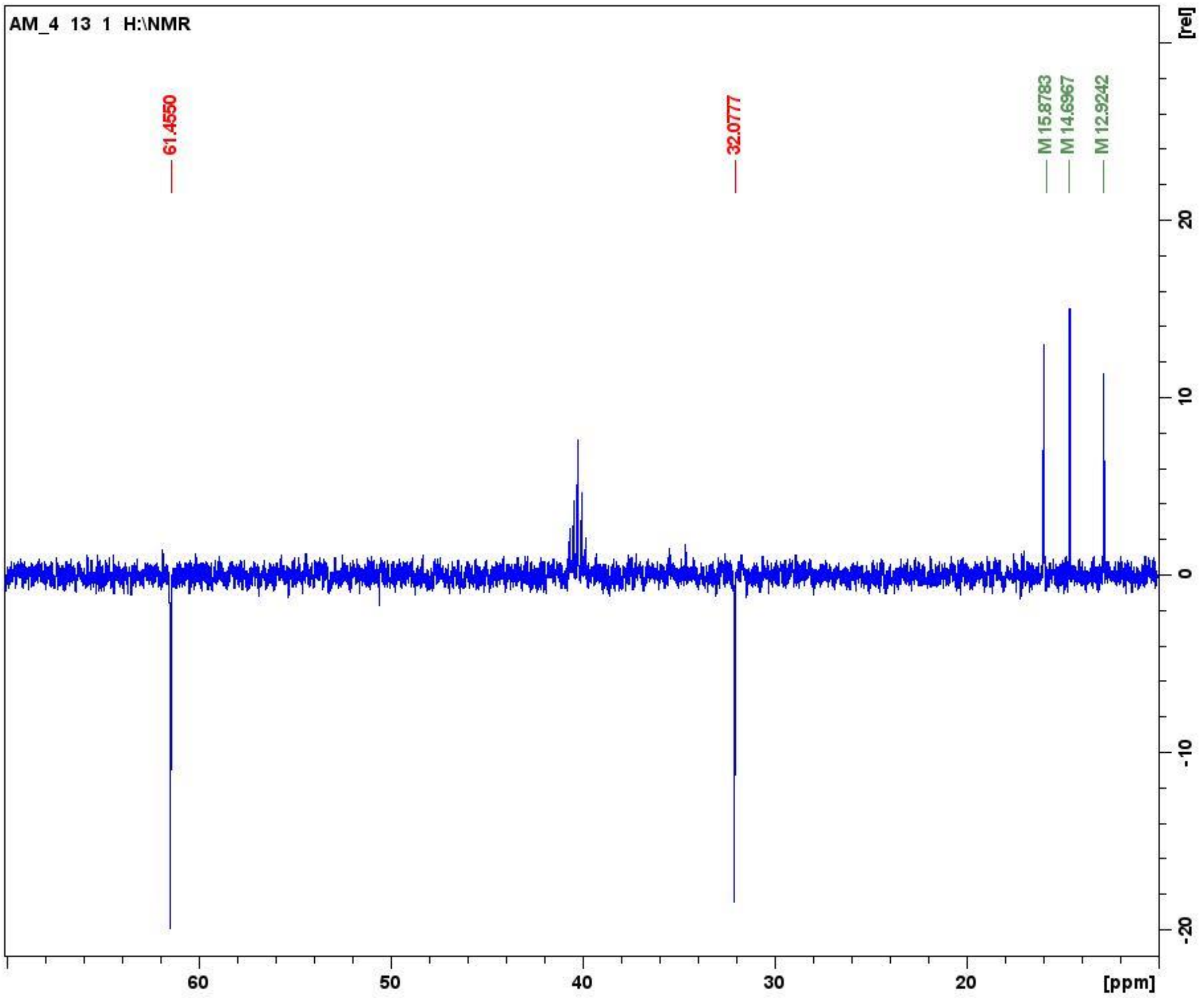

Figure S7-c DEPT-135 NMR spectrum of compound **4** in DMSO-d_6

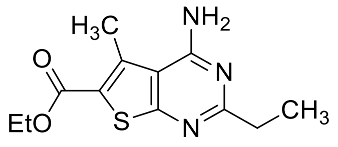

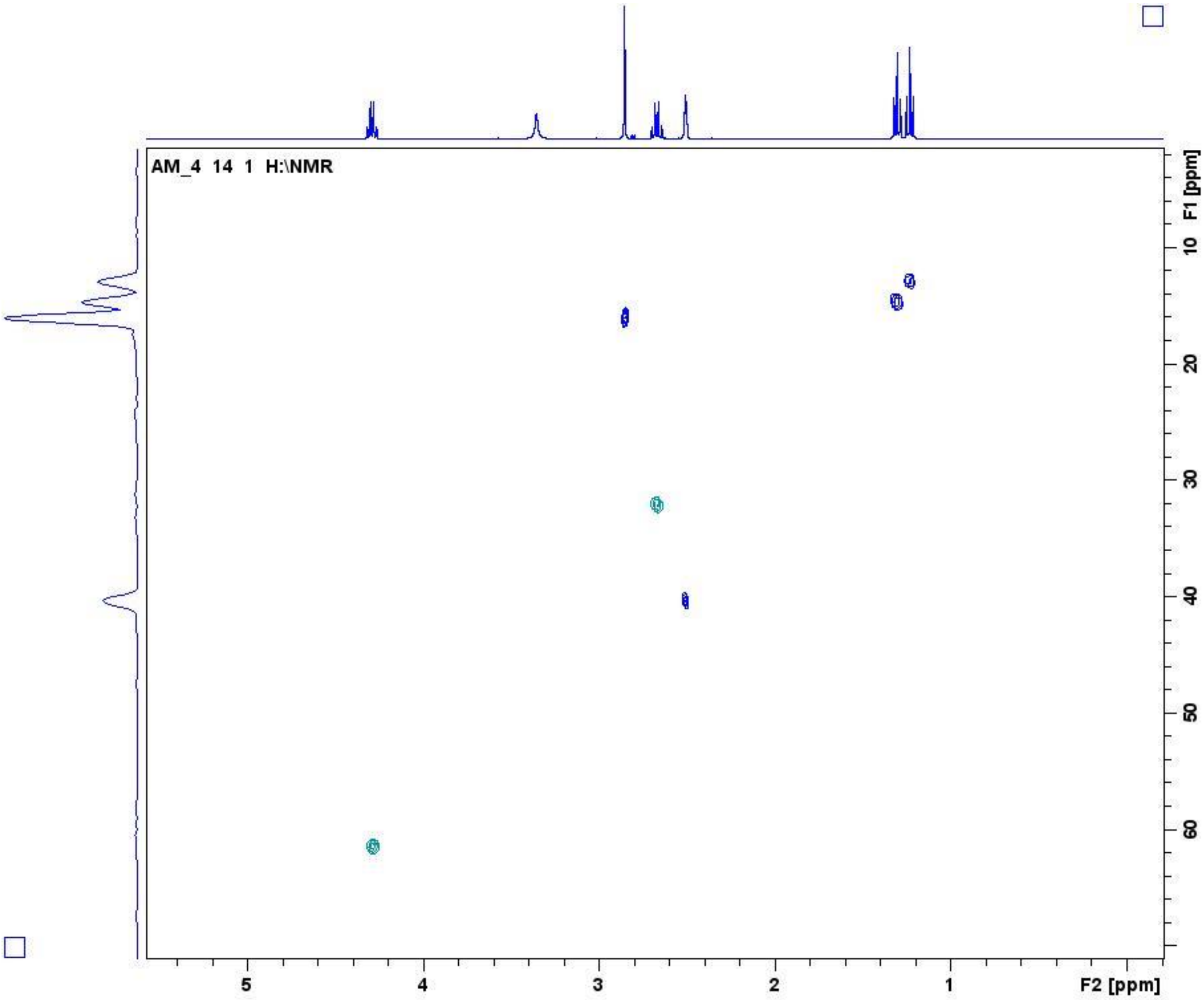

Figure S7-d HSQC NMR spectrum of compound **4** in DMSO-d_6

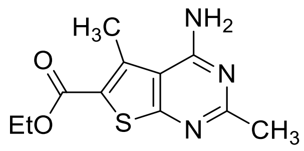

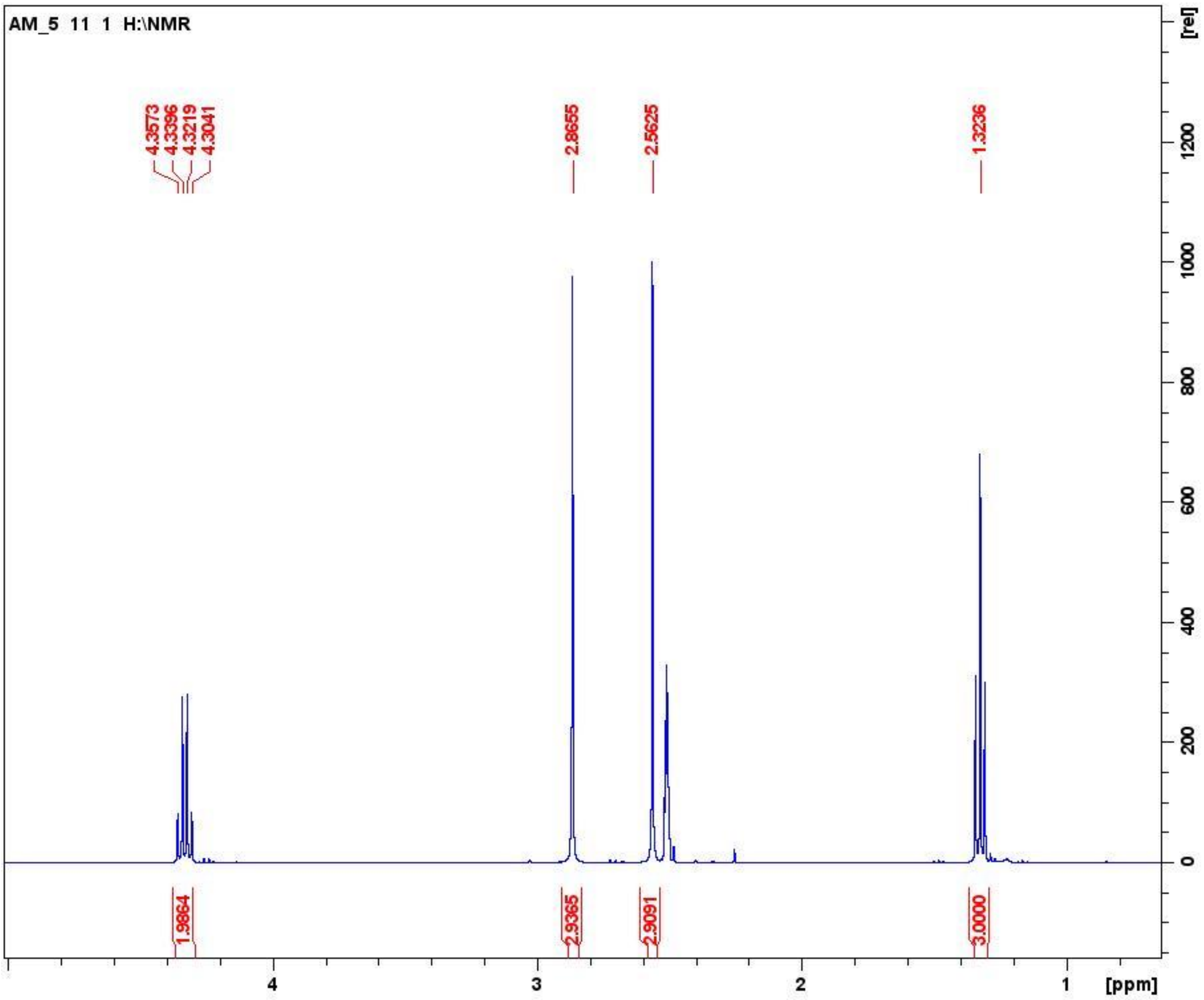

Figure S8-a ^{1}H NMR spectrum of compound **5** in DMSO-d_6

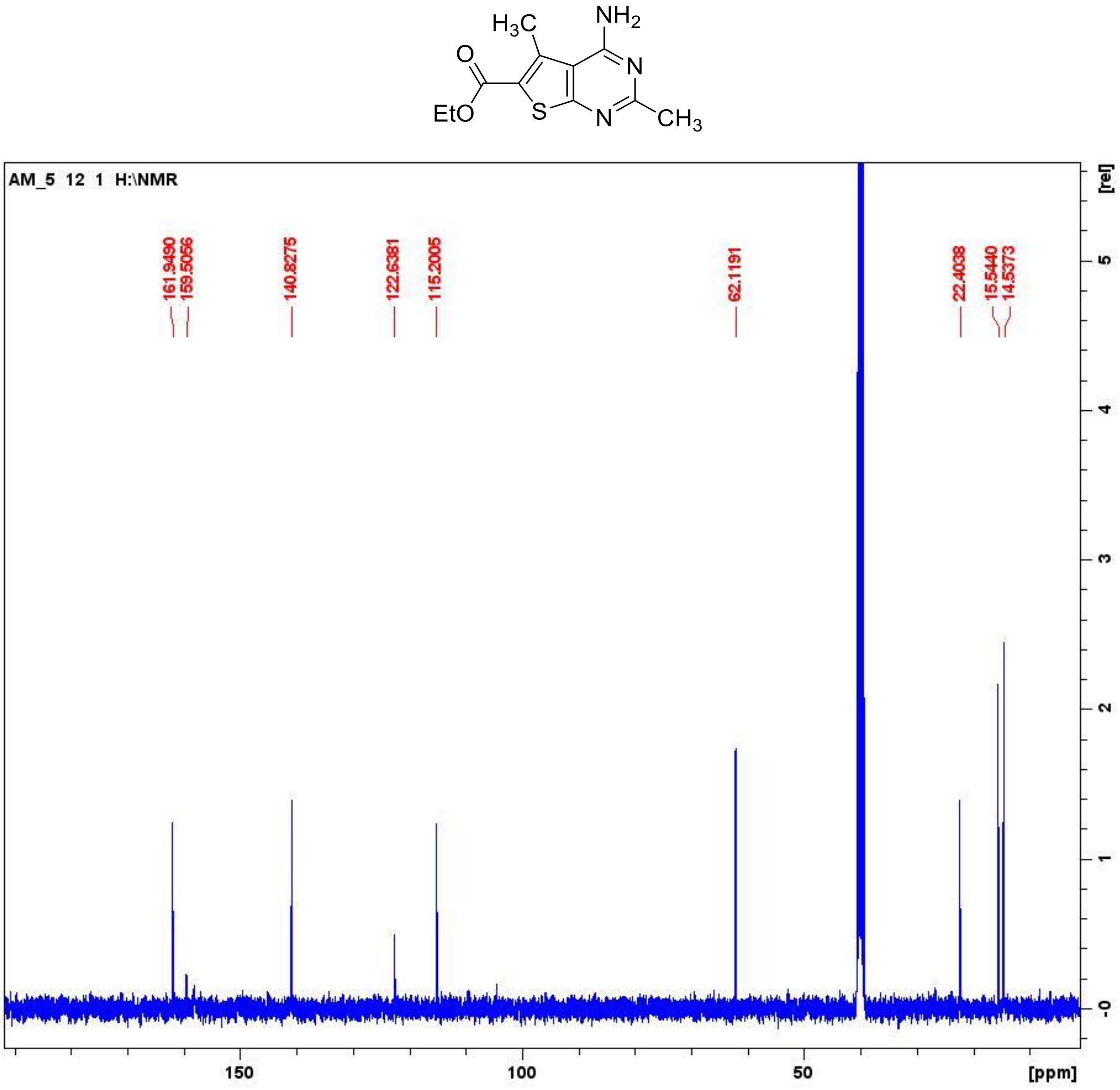

Figure S8-b ^{13}C NMR spectrum of compound **5** in DMSO-d_6

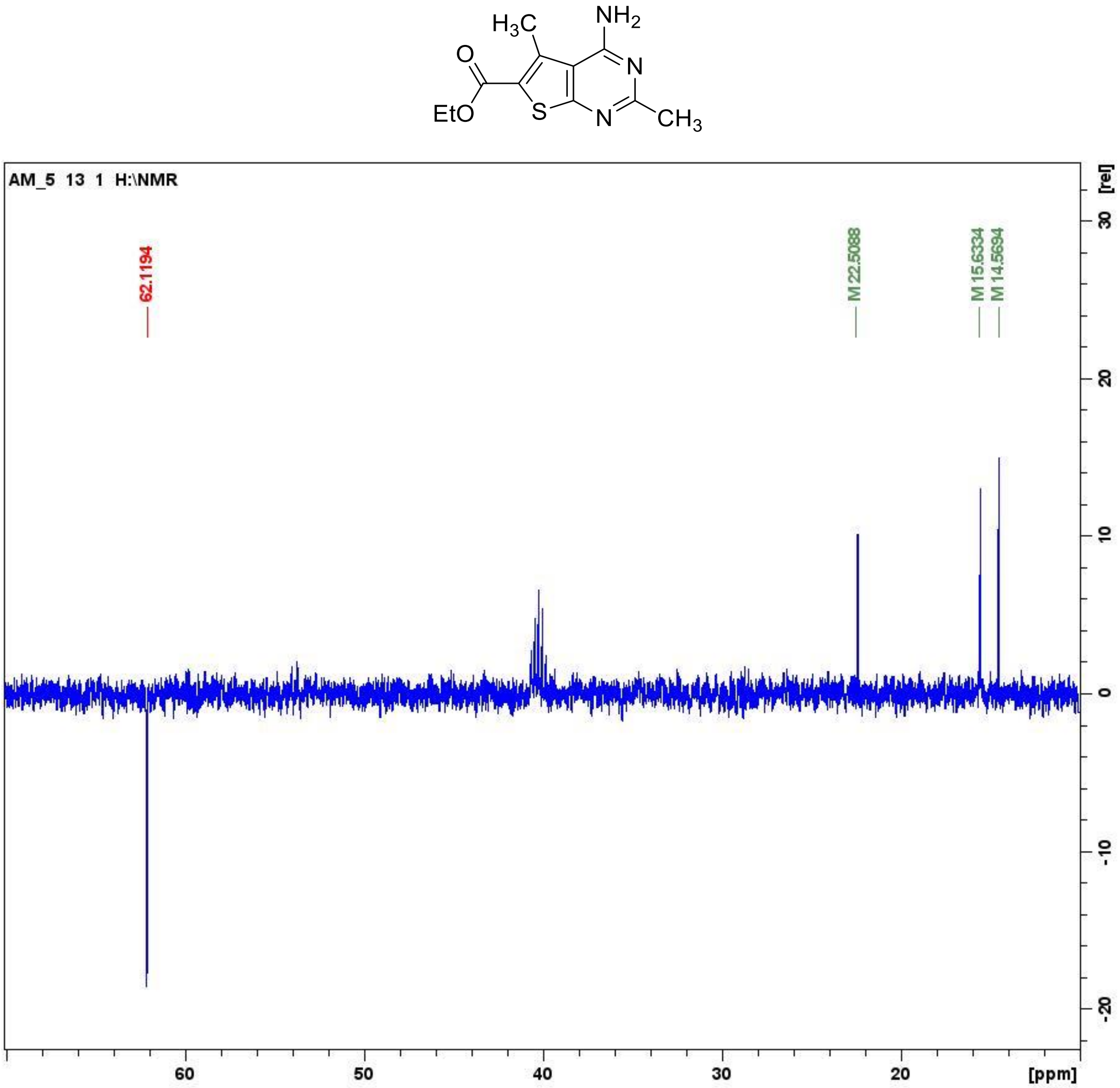

Figure S8-c DEPT-135 NMR spectrum of compound **5** in DMSO-d_6

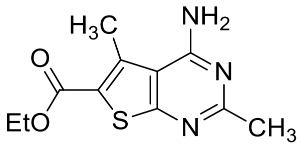

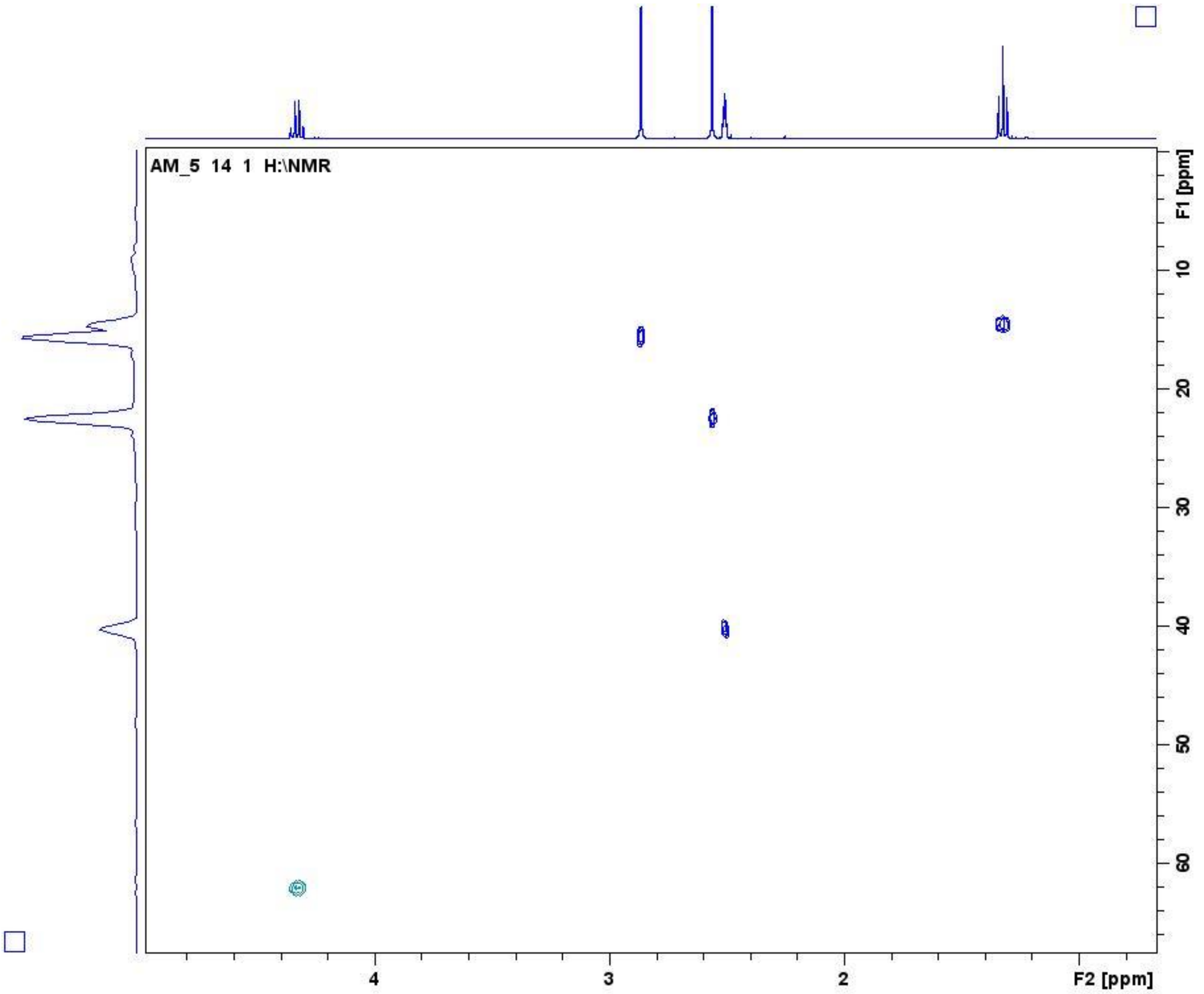

Figure S8-d HSQC NMR spectrum of compound **5** in DMSO-d_6

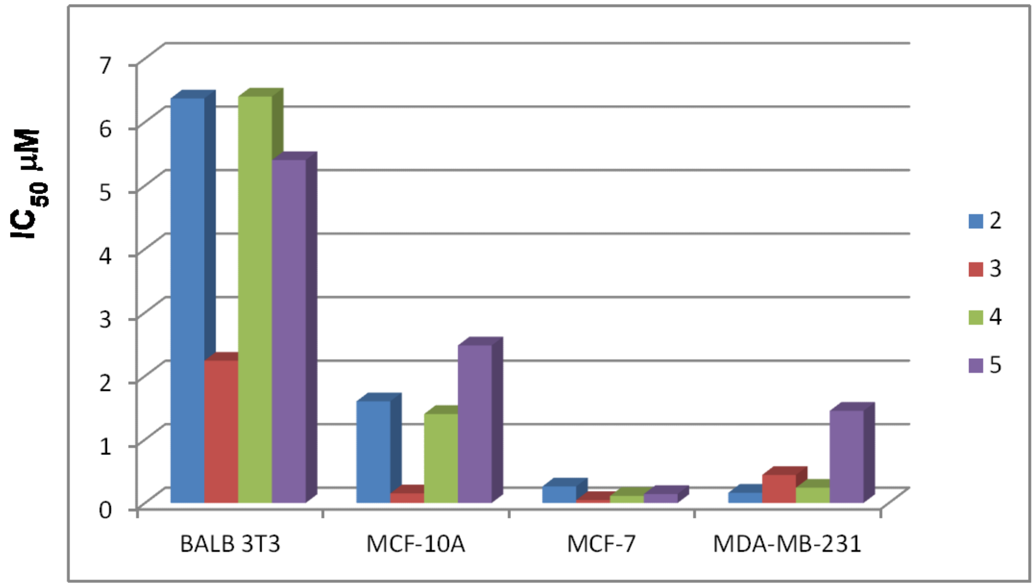

Figure S9 Cytotoxicity of compounds **2-5** against BALB 3T3, MCF-10A, MCF-7 and MDA-MB-231 cells

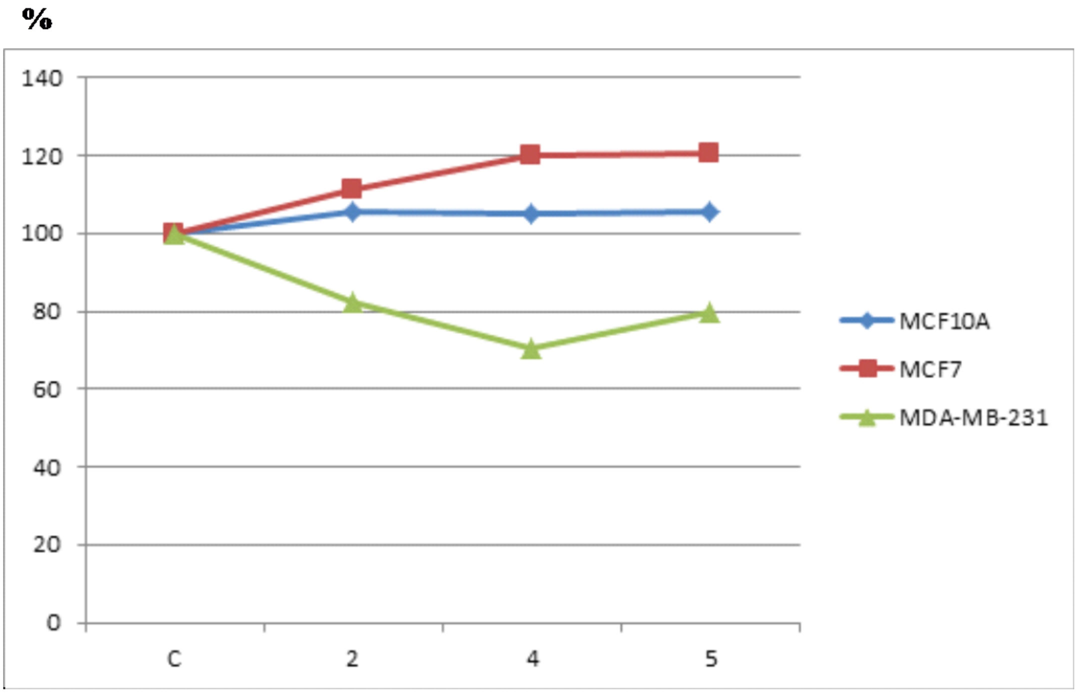

Figure S10 Cell cycle arrest – G1 phase

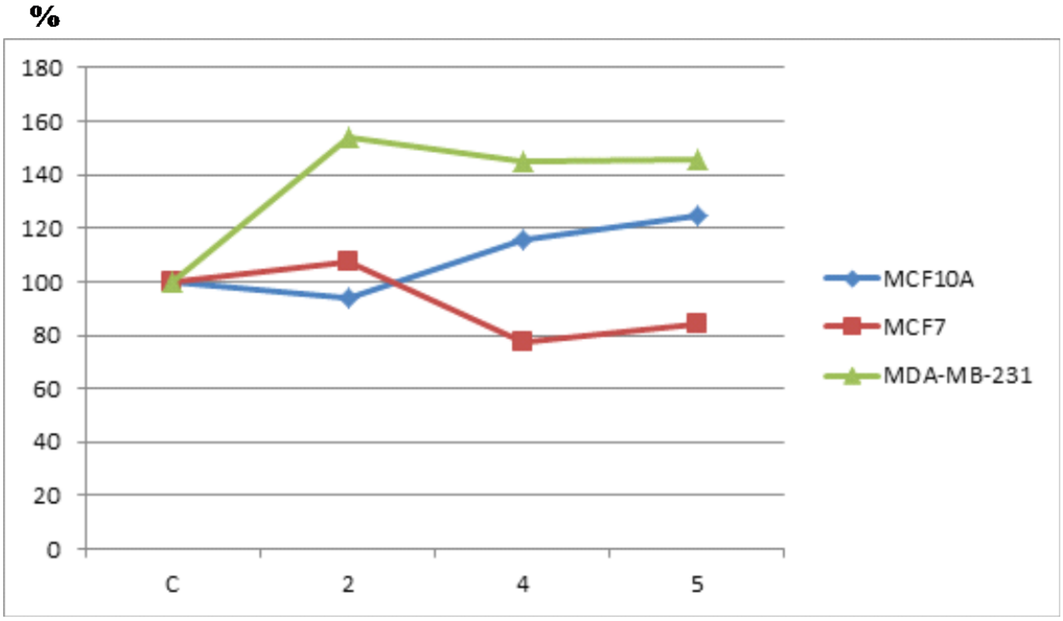

Figure S11 Cell cycle arrest – G2 phase

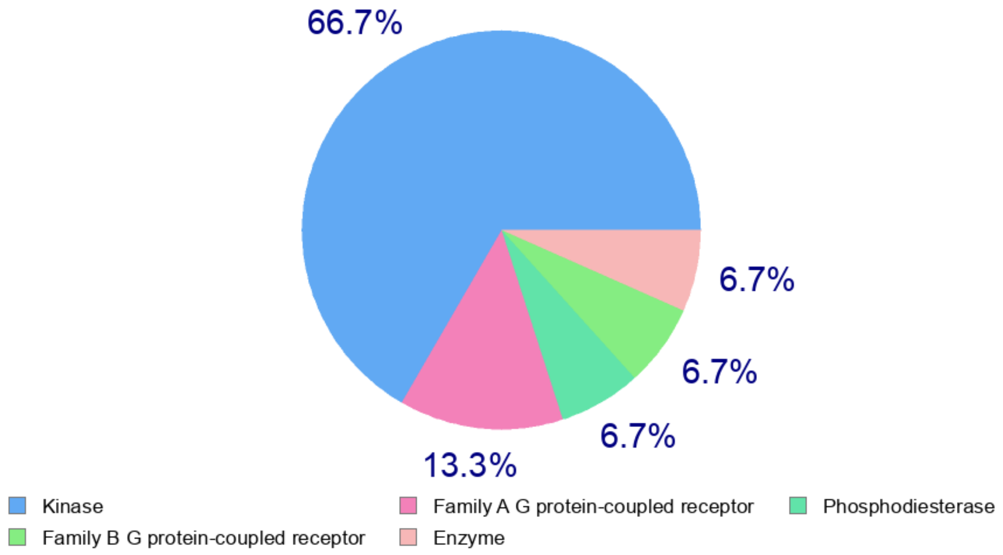

Figure S12 Predicted protein targets of compund **2**

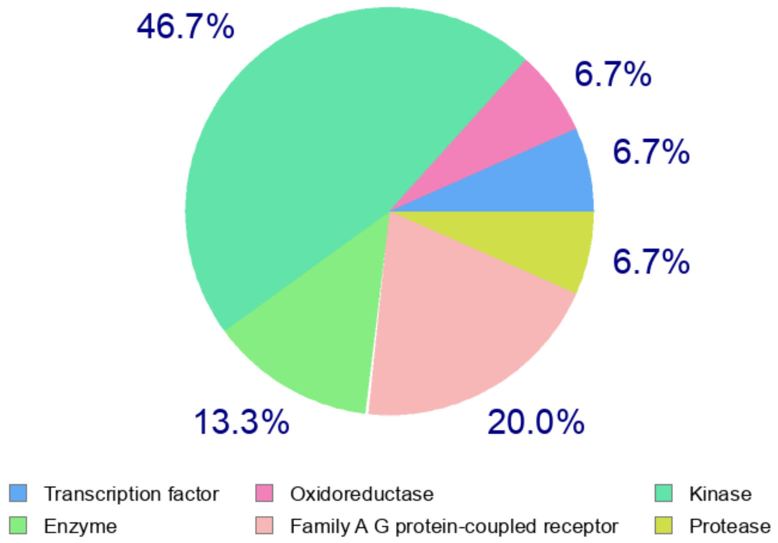

Figure S13 Predicted protein targets of compound **3**

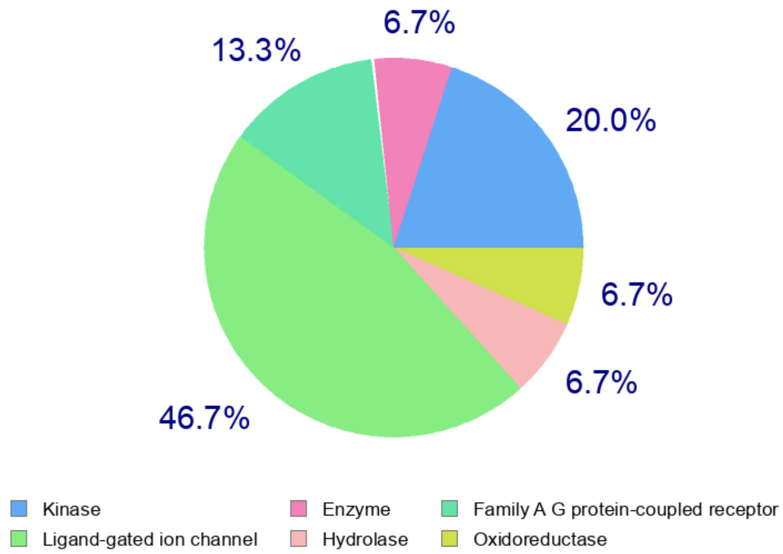

Figure S14 Predicted protein targets of compound **5**

Calculated lipophilicity of compound 2

Log $P_{o/w}$ (iLOGP)	2.56
Log $P_{o/w}$ (XLOGP3)	2.32
Log $P_{o/w}$ (WLOGP)	1.87
Log $P_{o/w}$ (MLOGP)	1.28
Log $P_{o/w}$ (SILICOS-IT)	3.28
Consensus Log $P_{o/w}$	2.26

Calculated lipophilicity of compound 3

Log $P_{o/w}$ (iLOGP)	2.82
Log $P_{o/w}$ (XLOGP3)	3.01
Log $P_{o/w}$ (WLOGP)	2.55
Log $P_{o/w}$ (MLOGP)	1.89
Log $P_{o/w}$ (SILICOS-IT)	3.92
Consensus Log $P_{o/w}$	2.84

Calculated lipophilicity of compound 4

Log $P_{o/w}$ (iLOGP)	2.63
Log $P_{o/w}$ (XLOGP3)	2.96
Log $P_{o/w}$ (WLOGP)	2.33
Log $P_{o/w}$ (MLOGP)	1.62
Log $P_{o/w}$ (SILICOS-IT)	3.39
Consensus Log $P_{o/w}$	2.59

Calculated lipophilicity of compound 5

Log $P_{o/w}$ (iLOGP)	2.44
Log $P_{o/w}$ (XLOGP3)	2.52
Log $P_{o/w}$ (WLOGP)	2.08
Log $P_{o/w}$ (MLOGP)	1.34
Log $P_{o/w}$ (SILICOS-IT)	3.02
Consensus Log $P_{o/w}$	2.28
